# Destruction of Tumors by Electricity

**Published:** 1865-12

**Authors:** 


### DESTRUCTION OF TUMORS BY ELECTRICITY.

Mr. Nelaton communicates a paper on the destruction of tumors by an electrolytic method, applicable to cases in which the tumor is deeply seated, and inaccessible to ordinary instruments. The case quoted by him was a naso pharyngian polypus, which was electro-chemically destroyed by inserting two platinum pins into its mass, and making them communicate with the poles of a Bunsen's pile, consisting of nine elements, sixteen centimeters in height by eight in diameter, and arranged for tension. The tumor was destroyed in six sittings, without any effusion of blood, and with very little pain.—*Chicago Med. Ex. from Cor. of Med. and Surg. Reporter.*

